# Cortical GABA in Subjects at Ultra-High Risk of Psychosis: Relationship to Negative Prodromal Symptoms

**DOI:** 10.1093/ijnp/pyx076

**Published:** 2017-08-19

**Authors:** Gemma Modinos, Fatma Şimşek, Jamie Horder, Matthijs Bossong, Ilaria Bonoldi, Matilda Azis, Jesus Perez, Matthew Broome, David J Lythgoe, James M Stone, Oliver D Howes, Declan G Murphy, Anthony A Grace, Paul Allen, Philip McGuire

**Affiliations:** 1 Department of Psychosis Studies, King’s College London; 2 Department of Forensic and Neurodevelopmental Sciences and Sackler Institute for Translational Neurodevelopment, King’s College London; 3 Institute of Psychiatry, Psychology & Neuroscience, King’s College London, United Kingdom; 4 Department of Psychiatry, Brain Center Rudolf Magnus, University Medical Center, Utrecht, The Netherlands; 5 CAMEO Early Intervention in Psychosis Service, Cambridgeshire and Peterborough NHS Foundation Trust, Cambridge, United Kingdom; 6 Department of Psychiatry, University of Cambridge, Cambridge, United Kingdom; 7 Department of Psychiatry, and Faculty of Philosophy, University of Oxford, Oxford, United Kingdom; 8 Oxford Health NHS Foundation Trust, Oxford, United Kingdom; 9 Department of Neuroimaging, Institute of Psychiatry, Psychology & Neuroscience, King’s College London, London, United Kingdom; 10 Department of Neuroscience, University of Pittsburgh, Pittsburgh, Pennsylvania; 11 Department of Psychology, University of Roehampton, London, United Kingdom

**Keywords:** psychosis, magnetic resonance spectroscopy, GABA, ultra-high risk of psychosis, negative symptoms

## Abstract

**Background:**

Whilst robust preclinical and postmortem evidence suggests that altered GABAergic function is central to the development of psychosis, little is known about whether it is altered in subjects at ultra-high risk of psychosis, or its relationship to prodromal symptoms.

**Methods:**

Twenty-one antipsychotic naïve ultra-high risk individuals and 20 healthy volunteers underwent proton magnetic resonance imaging at 3T. Gamma-aminobutyric acid levels were obtained from the medial prefrontal cortex using MEGA-PRESS and expressed as peak-area ratios relative to the synchronously acquired creatine signal. Gamma-aminobutyric acid levels were then related to severity of positive and negative symptoms as measured with the Community Assessment of At-Risk Mental States.

**Results:**

Whilst we found no significant difference in gamma-aminobutyric acid levels between ultra-high risk subjects and healthy controls (*P*=.130), in ultra-high risk individuals, medial prefrontal cortex GABA levels were negatively correlated with the severity of negative symptoms (*P*=.013).

**Conclusion:**

These findings suggest that gamma-aminobutyric acidergic neurotransmission may be involved in the neurobiology of negative symptoms in the ultra-high risk state.

## Introduction

Converging evidence from postmortem and preclinical studies indicates that dysfunction of the gamma-aminobutyric acidergic (GABAergic) neurotransmitter system plays a major role in the pathophysiology of schizophrenia (Marin, 2012). Postmortem research has demonstrated decreased mRNA expression of glutamic acid decarboxylase and reduced density of fast-spiking parvalbumin-positive interneurons in a corticolimbic circuitry involving the prefrontal cortex and the amygdala in schizophrenia (Lewis et al., 2005; Akbarian and Huang, 2006; Benes, 2010). Furthermore, animal models of psychosis suggest a link between disrupted cortical GABAergic function and dysregulation of subcortical dopaminergic signaling characteristic of the disorder (Grace, 2010). Such models propose that inhibitory disruption would underlie not only dopamine-dependent positive symptoms of psychosis, but would also influence other neural pathways (e.g., including the basolateral nucleus of the amygdala, or the medial prefrontal cortex [MPFC]) putatively involved in the development of the negative symptoms of psychosis (Grace, 2016). Moreover, the role of GABA in the development of psychosis is further supported by preclinical evidence that peripubertal (i.e., premorbid) pharmacological intervention on GABA-Aα5 receptors prevents schizophrenia-like GABA cell loss and blocks the development of psychosis-like features in adult rats (Du and Grace, 2013, 2016).

From animal and human postmortem studies, it may thus be hypothesized that cortical GABAergic function is reduced in schizophrenia and that this abnormality can be detected in the premorbid stages of the disorder (Modinos et al., 2015). However, recent meta-analytical evidence from human imaging studies using proton magnetic resonance spectroscopy (^1^H-MRS) did not show a significant difference in regional GABA levels between patients with schizophrenia and healthy volunteers (Egerton et al., 2017). Research in patients with schizophrenia is complicated by previous antipsychotic exposure and heterogeneity of clinical subgroups (Kegeles et al., 2012). In this context, studies in subjects at ultra-high risk (UHR) of developing psychosis are a useful resource to investigate neurobiological correlates of psychosis-like characteristics without confounds associated with the use of antipsychotics or illness chronicity on the imaging data. The 3 available MPFC GABA studies in UHR individuals have also presented mixed results, including increases (de la Fuente-Sandoval et al., 2016), decreases (Menschikov et al., 2016), and no differences (Wang et al., 2016) when compared with healthy controls. Nevertheless, heterogeneity of clinical subgroups is also a potential confounder in UHR studies (Fusar-Poli et al., 2016), and only 1 study to date investigated associations between GABA levels and severity of positive and negative symptoms in this group (de la Fuente-Sandoval et al., 2016). The present study sought to address these issues by using ^1^H-MRS in a homogenous sample of antipsychotic-naïve subjects at UHR of psychosis to test the hypotheses that: (1) GABA levels in the MPFC would be reduced in UHR subjects compared with healthy controls (Marin, 2012), and that (2) GABA levels would be inversely related to the severity of positive and negative prodromal symptoms (Grace, 2016).

## Methods

Procedures were approved by the Research Ethics Committee of King’s College London and South London and Maudsley NHS Trust. All participants provided informed consent.

Twenty-one UHR individuals and 20 healthy volunteers, all males aged 18to 30 years, were included. UHR psychopathology was assessed using the Community Assessment of At-Risk Mental States (CAARMS) (Yung et al., 2005). UHR inclusion criteria required the presence of one or more of the following: (1) attenuated psychotic syndrome, (2) a brief psychotic episode of <1 week duration that spontaneously remits without antipsychotic medication/hospitalization, and (3) trait vulnerability (schizotypal personality disorder or a first-degree relative with psychosis) plus a marked decline in psychosocial functioning. Healthy control subjects were recruited from the local community. They were excluded if they had a personal or familial history of psychiatric disorder, neurological illness, or drug/alcohol dependence based on the DSM-V (American Psychiatric Association, 1994). Current/past medication use and current/past use of tobacco and cannabis was assessed using a semistructured interview adapted from the Early Psychosis Prevention and Intervention Centre Drug and Alcohol Assessment Schedule (http://www.eppic.org.au). All subjects were safe for MRI, had an IQ in the normal range as assessed using the Wechsler Adult Intelligence Scale-III (Velthorst et al., 2013), and were antipsychotic-naïve, and none were taking benzodiazepines.

**Figure 1.**
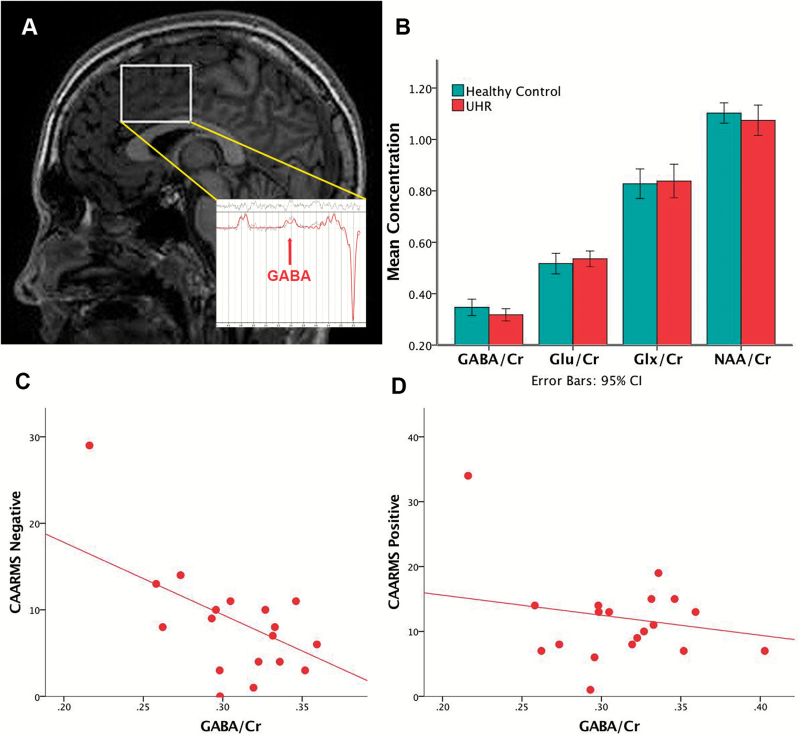
(A) Voxel placement on medial prefrontal cortex (MPFC) and representative sample ^1^H-MRS spectra. (B) MPFC metabolite levels by group. (C) Scatterplot of the significant association between CAARMS negative symptom severity and GABA/Cr levels (*ß* = -.556, *t* = -2.761, *R*^*2*^ = .310, *P* = .013). (D) Scatterplot of the nonsignificant associations between CAARMS positive symptom severity and GABA/Cr levels (*P* = .298). Cr, creatine; Glu, glutamate; Glx, glutamate + glutamine; NAA, N-Acetyl-aspartate; UHR, ultra-high risk of psychosis.

Subjects underwent ^1^H-MRS on a General Electric Signa HDx TwinSpeed 3T scanner at the Centre for Neuroimaging Sciences, Institute of Psychiatry, Psychology & Neuroscience (King’s College London). GABA levels were obtained from the MPFC using MEGA-PRESS, which incorporates a standardized chemically selective suppression water suppression routine (TE=68 milliseconds, TR=2000 milliseconds). For each acquisition, unsuppressed water reference spectra (16 averages) were also acquired. Shimming was optimized, with auto-prescan performed twice before each scan. The region of interest in the MPFC was prescribed from the midline sagittal localizer, and the center of the 40-×35-×20-mm region of interest was placed above the middle section of corpus callosum (Figure 1A). Spectra were analyzed using LCModel 6.3-1L with the basis set provided by its author (Provencher, 2016), which contained the metabolites GABA, glutamine, glutamate, Glx (glutamate + glutamine), and N-acetyl-aspartate (NAA). We used Cramer-Rao minimum variance bounds (CRLB)>20% as reported by LCModel, which are estimates of fit of the metabolite peaks, and signal-to-noise ratio <8 to exclude poorly fitted metabolite peaks from statistical analysis (Mouchlianitis et al., 2016; Provencher, 2016). Data from all 41 participants in the present study met these criteria. Metabolites were expressed as ratios relative to the synchronously acquired creatine signal from the unedited MEGA-PRESS spectra. This is a well-established normalization procedure in clinical ^1^H-MRS studies that has been extensively used in previous studies of MPFC GABA levels in patients with schizophrenia (Goto et al., 2009; Ongur et al., 2010; Kegeles et al., 2012; Marsman et al., 2014) and UHR subjects (Marenco et al., 2016; Menschikov et al., 2016).

Analysis of demographic and metabolite data was performed with SPSS 24. Group differences were tested using independent-sample *t* tests, and significant effects are reported at *P* <.05. Associations between GABA/Cr levels and severity of CAARMS positive and negative symptoms were assessed with linear regression, and results were Bonferroni-corrected at *P*<.025. In line with previous studies (e.g., Kegeles et al., 2012; de la Fuente-Sandoval et al., 2016), exploratory analyses on the other metabolites in the spectra (Glu/Cr, Glx/Cr, and NAA/Cr) were conducted for completion but will not be discussed. These analyses explored: (1) group differences using *t* tests, (2) associations with CAARMS positive and negative symptoms using linear regression, and (3) correlations between GABA/Cr and the other metabolites using Pearson’s product-moment correlation (Bonferroni-corrected at *P*<.05/3). As field strengths of 4T or more are needed to measure glutamine accurately, exploratory analysis of this metabolite was not performed (Snyder and Wilman, 2010). Finally, Pearson’s product-moment correlation was used to examine potential associations between GABA levels and age as well as cigarette use in UHR subjects, and Mann Whitney-U test was used to examine potential group effects between UHR with and without current or past cannabis use.

## Results

Table 1 summarizes participant characteristics and metabolite values. All UHR subjects met Attenuated Psychosis Syndrome criteria.

**Table 1.** Participants’ Characteristics and Spectral Data

	HC (n = 20)	UHR (n = 21)	HC vs UHR	
	Mean (SD, range)	Mean (SD, range)	Statistic	P
Age (y)	23.7 (2.7, 20–28)	22.2 (3.0, 18–29)	*t* = 1.613	.111
Estimated IQ	117.1 (10.4, 95–132)	110.0 (12.5, 75–128)	*t* = 1.736	.092
CAARMS positive	-	11.9 (6.7, 1–34)	-	-
CAARMS negative	-	8.4 (6.4, 0–29)	-	-
Tobacco (cigarettes/d)	-	4.75 (6.7)	-	-
Cannabis now (yes/no)	-	11/12	-	-
Cannabis ever (yes/no)	-	16/4	-	-
SNR	21.6 (2.8)	21.7 (2.80)	-.076	.939
Line width	6.2 (1.8)	6.4 (1.28)	-.284	.778
GABA/creatine	.4 (.1)	.3 (.05)	1.546	.130
GABA % CRLB	5.5 (1.0)	5.9 (1.4)	-1.052	.300
Glutamate/creatine	.5 (.1)	5.5 (.1)	-.769	.446
Glutamate % CRLB	7.6 (1.5)	6.6 (1.5)	2.001	.053
Glx/creatine	.8 (.1)	.8 (.1)	-.255	.800
Glx % CRLB	5.6 (1.4)	5.2 (1.5)	.835	.409
N-acetyl-aspartate/creatine	1.1 (.1)	1.1 (1.1)	.823	.415
N-acetyl-aspartate % CRLB	1.15 (.37)	1.63 (1.34)	-1.512	.146

Abbreviations: CAARMS, Clinical Assessment for At-Risk Mental States; CRLB, Cramer Rao Lower Bounds; GABA, gamma-aminobutyric acid; Glx, glutamate + glutamine ratio; HC, healthy control subjects; SNR, signal to noise ratio; UHR, ultra-high risk subjects.

### GABA Levels

There were no significant differences in the creatine-scaled GABA levels between UHR subjects and healthy controls (*P=*.130). Exploratory analysis of the other metabolites in the spectra also showed no significant differences between groups corrected for multiple comparisons (all *P* >.017) (Table 1; Figure 1B).

Within the UHR group, MPFC GABA/Cr levels were inversely associated with the severity of negative symptoms (*ß*=-.556, *t*=-2.761, *P=*.013, significant after Bonferroni correction at *P*<.025), but there was no relationship with positive symptoms (*ß* =-.245, *t*=-1.071, *P=*.298) (Figure 1C–D).

MPFC GABA/Cr levels were not significantly associated with age (*r*=-.027, *P=*.908), cigarette use (*r*=-.195, *P=*.410), or differed in UHR subjects with current or past cannabis use compared with those without (current use: *U*=38.0, *Z*=-1.723, *P*=.085; past use: *U*=22.0, *Z*=-.945, *P* =.345). Groups did not differ in spectral quality (CRLB, *P*=.484; SNR, *P*=.939; linewidth, *P*=.778) (Table 1). Follow-up clinical data revealed that 3 of the 20 UHR subjects developed psychosis at a mean follow-up time o 18 months. Exploratory analyses removing these subjects rendered the correlation between GABA/Cr and CAARMS negative symptoms no longer significant (ß=-.377, t=-1.578, *P*=.135), suggesting that the association was driven by those individuals who went on to develop a psychotic disorder.

### Other Metabolites

Exploratory analysis showed no significant associations between levels of the other metabolites in the voxel and positive or negative symptom severity (Glu/Cr and CAARMS positive: *ß*=-.159, *t*=-.684, *P*=.503; Glu/Cr and CAARMS negative: *ß*=-.297, *t*=-1.283, *P*=.217; Glx/Cr and CAARMS positive: *ß*=-.109, *t*=-.463, *P*=.649; Glx/Cr and CAARMS negative: *ß*=-.096, *t*=-.400, *P*=.694; NAA/Cr and CAARMS positive: *ß*=-.173, *t*=-.746, *P*=.465; NAA/Cr and CAARMS negative: *ß*=-.034, *t*=-.142, *P* =.889). None of these metabolites were significantly correlated with age (Glu/Cr: *r*=-.296, *P*=.192; Glx/Cr: *r*=-.421, *P*=.058; NAA/Cr: *r*=.342, *P*=.130). Finally, Pearson’s product-moment correlation showed that in healthy controls, GABA/Cr was positively associated with NAA/Cr (*r* =.516, *P*=.020), while in UHR individuals, GABA/Cr was positively associated with Glu/Cr (*r*=.460, *P*=.041). However, these associations did not survive Bonferroni correction, or significantly differed between the groups (GABA and NAA: *z*=1.7, *P*=.089; GABA and Glu: *z*=-.69, *P*=.490).

## Discussion

We did not find evidence that cortical GABA levels (creatine-scaled) in subjects at UHR of psychosis differed from those in healthy controls. The 3 previous GABA MRS studies in UHR subjects reported either increased (de la Fuente-Sandoval et al., 2016), decreased (Menschikov et al., 2016), or no difference from healthy controls (Wang et al., 2016). Although the location of the ^1^H-MRS voxel in those previous UHR studies was more ventrally placed within the MPFC than in our study, another recent study in unaffected relatives using an overlapping voxel to ours did find a significant decrease in the relatives (Marenco et al., 2016). However, these were asymptomatic individuals at genetic high risk as opposed to a sample of subjects with an attenuated psychosis syndrome. Potential sources of variability may thus relate to voxel placement and to the nature of the high-risk sample under study. Future studies should consider standardizing voxel placement or using multiple rather than single voxels (Duyn et al., 1993). Allowing GABA quantification from both dorsal and ventral MPFC in the same individuals would help elucidate region-specific effects in people at increased risk for psychosis and clarify whether GABA function is relatively uncompromised in more dorsal MPFC areas compared with healthy individuals. Regarding the nature of the high-risk samples recruited to different studies, the UHR category is heterogeneous with respect to both inclusion criteria and clinical outcomes, and the neuroimaging findings in a sample may vary depending on its composition (Fusar-Poli et al., 2016). In this context, our study expands the previous literature by showing results from a homogeneous sample of UHR individuals who all fell under attenuated psychosis syndrome criteria. Prospective studies in similarly homogenized UHR samples are needed to further clarify whether GABAergic dysfunction can be reliably detected in this group, whether the MPFC subregions affected are predominantly ventral, and whether alterations in GABA levels are identifiable with ^1^H-MRS in UHR individuals who are destined to develop a psychotic disorder. Nevertheless, it is worth noting that a recent meta-analysis of ^1^H-MRS studies in schizophrenia did not find a significant effect in GABA levels (Egerton et al., 2017), suggesting a lack of convergence with predictions from animal and postmortem studies. This might relate to the divergent nature of the measurements across disciplines. While preclinical and postmortem studies suggest that the GABAergic abnormality refers to parvalbumin-positive interneurons (Marin, 2012), ^1^H-MRS assesses total tissue concentrations and as such it is likely to not be restricted to a particular GABA cell type. Future translational animal and human work measuring GABA levels in homolog regions across species may be able to comprehensively delineate the molecular pathway linking GABAergic dysfunction to the expression of schizophrenia-like characteristics, including GABA measurements in other anatomical regions such as the hippocampus, amygdala, and thalamus.

Although preclinical models would primarily predict that cortical GABA dysfunction leads to positive psychotic symptoms through a hyper-responsive dopamine system arising from a glutamatergic dysregulation, our regression analysis did not reveal an association between GABA and positive symptoms. However, we observed that GABA levels were significantly inversely associated with the severity of negative symptoms. This finding is of interest, as the pathophysiological basis of negative symptoms is unclear (Salamone et al., 2015), and it merits replication in a larger sample. Previous studies had not found a significant association between MPFC GABA levels and severity of positive or negative symptoms in UHR subjects (de la Fuente-Sandoval et al., 2016) or in unmedicated patients with a first episode of psychosis (Kegeles et al., 2012). Nevertheless, as mentioned above, effects of anatomical location of the MEGA-PRESS acquisition and study sample composition may be at play. Furthermore, although this cross-sectional study was not designed to examine longitudinal effects, the correlation appeared to be driven by UHR individuals who went on to develop a psychotic disorder. Preclinical models have proposed circuit-based approaches to the development of psychosis-like behaviors (Lisman et al., 2008), and it has been recently postulated that the positive, negative, and disorganized dimensions of schizophrenia may originate from disruption of multiple, interconnected circuits involving GABAergic dysfunction and converging on hippocampal hyperactivity (Grace, 2016). Hence, larger multimodal imaging studies in UHR subjects investigating associations between, for example, GABA levels and hippocampal activity in relation to clinical symptomatology and outcomes are warranted to expand the present findings.

Strengths of the current report include a homogenous UHR sample as determined by trained clinicians, a well-matched control group, and a validated method (MEGA-PRESS) to quantify water-scaled GABA concentrations at 3T. A limitation intrinsic to all MEGA-PRESS studies is that the GABA signal contains some contribution from macromolecules, that is, diverse proteins and lipids, but it is unlikely that the macromolecule contribution would differ across groups. Furthermore, the results should be considered in the context of a sample of male participants and as such may not generalize to all individuals with an attenuated psychosis syndrome.

In conclusion, if there is any alteration in GABA levels measurable with in vivo ^1^H-MRS in the MPFC in people at UHR of psychosis, it is small and difficult to detect even with a homogeneous sample of antipsychotic-naïve individuals. Nevertheless, our data provides a direct link between GABAergic levels and prodromal negative symptoms, which warrants replication in larger samples.

## Funding

This work was supported by a Wellcome Trust Programme Grant to P.M. (grant no. 091667, 2011) and a Wellcome Trust Programme Grant to D.G.M. (grant no. 091300/Z/10/Z). D.G.M. was also supported by the Dr Mortimer D. Sackler Foundation. G.M. is funded by the Wellcome Trust and the Royal Society (Sir Henry Dale Fellowship).

## Interest Statement

Dr Grace receives consulting fees from Johnson & Johnson, Lundbeck, Pfizer, GSK, Merck, Takeda, Dainippon Sumitomo, Otsuka, Lilly, Roche, Asubio, and Abbott; and receives research funding from Lundbeck, Lilly, Autifony, Alkermes, and Johnson & Johnson. Dr Howes has received investigator-initiated research funding from and/or participated in advisory/speaker meetings organized by Astra-Zeneca, Autifony, BMS, Eli Lilly, Heptares, Jansenn, Lundbeck, Lyden-Delta, Otsuka, Servier, Sunovion, Rand, and Roche. Neither Dr Howes nor his family have been employed by or have holdings/a financial stake in any biomedical company. There are no other interests from any of the other coauthors.
